# Clinical performance of metagenomic next-generation sequencing for diagnosis of invasive fungal disease after hematopoietic cell transplant

**DOI:** 10.3389/fcimb.2024.1210857

**Published:** 2024-03-25

**Authors:** Xiaoying Zhang, Lingfeng Zhang, Yun Li, Na Wang, Yicheng Zhang

**Affiliations:** ^1^Department of Hematology, Tongji Hospital, Tongji Medical College, Huazhong University of Science and Technology, Wuhan, Hubei, China; ^2^Key Laboratory of Organ Transplantation, Ministry of Education, NHC Key Laboratory of Organ Transplantation, Key Laboratory of Organ Transplantation, Chinese Academy of Medical Sciences, Wuhan, China; ^3^Immunotherapy Research Center for Hematologic Diseases of Hubei Province, Wuhan, Hubei, China

**Keywords:** metagenomic next-generation sequencing, invasive fungal disease, hematopoietic stem cell transplantation, immunosuppression, diagnosis

## Abstract

**Background:**

Timely diagnosis and appropriate antifungal therapy are critical for improving the prognosis of patients with invasive fungal disease (IFD) after hematopoietic stem cell transplantation (HSCT). We evaluated the performance of metagenomic next-generation sequencing (mNGS) and conventional microbiological testing (CMT), as well as the diagnosis, therapeutic management, and outcomes of IFD after HSCT.

**Methods:**

We retrospectively studied 189 patients who underwent HSCT and were considered at risk for IFD. In total, 46 patients with IFD were enrolled in this study. The IFD consensus was followed for classifying IFD incidents.

**Results:**

Forty-six patients were diagnosed with proven/probable (n = 12), possible (n = 27), and undefined (n = 7) IFD. *Aspergillus* was the most commonly detected fungal genus. *Mucormycosis* was found in 15 patients; two had *Aspergillus*, and one had *Candida* infections. Compared to CMT, mNGS significantly reduced the time required to identify pathogens (*P* = 0.0016). mNGS had a much higher sensitivity than CMT (84.78% vs. 36.96%; *P* < 0.0001). A total of 76.09% of patients received antifungal prophylaxis during fungal infections. All *Pneumocystis* infections occurred later than 100 days after transplantation. Among patients with *Pneumocystis* infection, 71.43% occurred following sulfonamide withdrawal, and subsequent treatment with sulfonamide alone or in combination with other drugs was effective. Based on the empirical antifungal treatment, the dosages, modes of administration, frequency of administration, or antifungal of 55.26% of the patients were changed according to the mNGS results. The 4-year overall survival rate of patients diagnosed with IFD after transplantation was 71.55% (95% CI, 55.18%–85.82%). Hypoproteinemia and corticosteroid use are independent risk factors for IFD.

**Conclusion:**

mNGS, which has a high sensitivity and a short detection time, aids in the diagnosis and prognosis of pathogenic fungi. As a powerful technology, mNGS can influence treatment decisions in patients with IFD following HSCT.

## 1 Introduction

Invasive fungal disease (IFD) is a common complication after hematopoietic stem cell transplantation (HSCT) and a leading cause of transplant-related mortality (Pagano et al., 2007; Sun et al., 2015). Patients experience conditioning regimens, agranulocytosis, impaired mucosal barrier, use of central venous catheterization, graft-versus-host disease (GVHD), immunosuppression, and delayed immune reconstitution, which all significantly increase the risk of IFD (Kuster et al., 2018; Sun et al., 2021). Advances in microbiological techniques and antifungal drugs have resulted in improvements in IFD diagnosis and treatment (Akan et al., 2013; Tissot et al., 2017; Chinese Association, H and Chinese Invasive Fungal Infection Working, G, 2020; Ruhnke et al., 2020; Chinese Society Of Hematology and Chinese Medical Association, Antimicrobial Infection Branch, 2023). The specific symptoms and adequate diagnostic procedures for IFD remain lacking, resulting in delayed diagnosis and treatment of IFD and a poor prognosis of patients. Early detection of IFD and prompt initiation of appropriate treatment are critical factors in the survival of patients following HSCT (Puerta-Alcalde and Garcia-Vidal, 2021)

Standard diagnostic techniques are clinically challenging for IFD diagnosis because of their invasiveness, long detection period, and lack of sensitivity, specificity, and species identification (Ostrosky-Zeichner, 2012). The gold standard for diagnosing IFD is culture-based testing; unfortunately, sterile specimens frequently necessitate potentially invasive procedures (Donnelly et al., 2020). Moreover, serum biomarkers, including galactomannan (GM) and 1,3-β-D-glucan (BDG), are the adjunct to clinical diagnosis of IFD (Guo et al., 2010; Lu et al., 2011a; Li et al., 2015; Donnelly et al., 2020). However, GM and BDG cannot detect all fungal pathogens, and their diagnostic efficacy in HSCT varies (Ullmann et al., 2018; Warris et al., 2019). According to the European Conference on Infections in Leukemia guidelines, serum BDG can help diagnose Pneumocystis jirovecii pneumonia (Maertens et al., 2016). However, GM performance remains significantly lower in non-neutropenic patients and/or those receiving prophylactic therapy (Wu et al., 2021). Radiographic findings may aid in the diagnosis of fungal lung lesions in patients with fever and neutropenia (FN). Nonspecific observations, on the other hand, often lead to overdiagnosis and incorrect treatment. Although PCR-based technologies have been shown to be effective in the identification of some fungal pathogens, false-positive and false-negative results limit their widespread application (Lu et al., 2011b; Trubiano et al., 2016; Ala-Houhala et al., 2018).

Metagenomic next-generation sequencing (mNGS) is a promising culture-independent technique that has been extensively used to diagnose infections (Gabaldón, 2019; Irinyi et al., 2020). mNGS may detect many types of microorganisms in a microbial sample both rapidly and concurrently (Barreda-García et al., 2018) while also recognizing non-culturable microbes (Yang et al., 2022). The application of mNGS to identify fungal infections has recently increased (Alonso et al., 2018; Shivaji et al., 2019; Chien et al., 2022). However, there have been relatively few investigations on the use of mNGS for diagnosis in patients with IFD following HSCT, and its clinical application has not been standardized. Owing to the severity and specificity of IFD after HSCT, efforts to optimize pathogen detection technologies are important for a favorable prognosis. This study examined the efficacy of standard mNGS technology in detecting infections in patients with IFD after HSCT by comparing the diagnostic performance of mNGS and conventional microbiological testing (CMT). In addition, we describe IFD diagnoses, survival, and risk factors for IFD following HSCT.

## 2 Material and methods

### 2.1 Study design and patients

**Figure 1:**
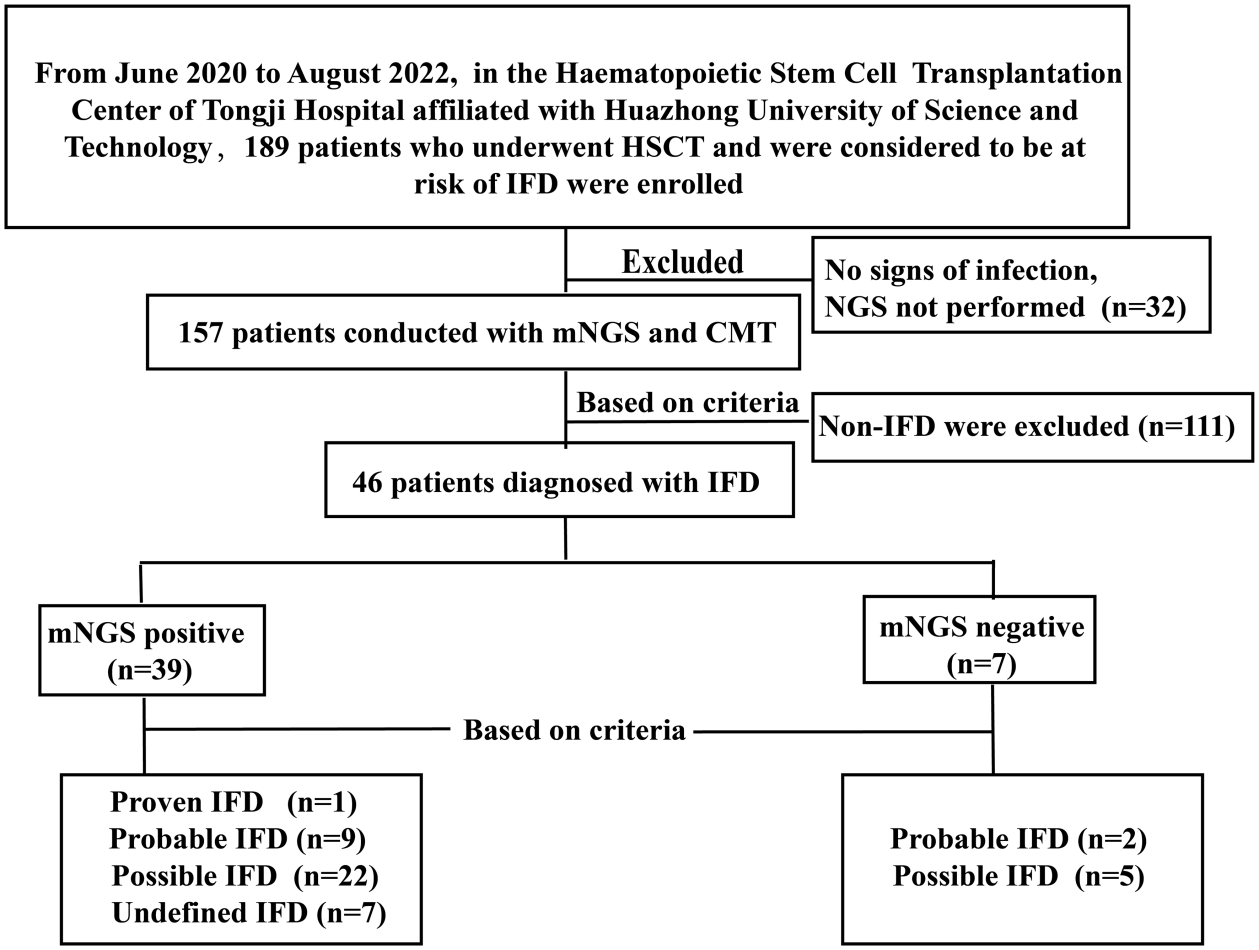
Flow diagram of the patients included in the study.

We retrospectively studied 189 patients who underwent HSCT and were considered at risk of IFD at the Hematopoietic Stem Cell Transplantation Center, Tongji Hospital, affiliated with Huazhong University of Science and Technology, between June 2020 and October 2022. At least one of the following enrollment criteria was present: (1) prolonged fever with neutropenia (FN) after broad-spectrum antibiotics (≥ 96 h), (2) FN recurrence, or (3) anomalous conditions to consider fungal infections. Ultimately, 46 patients with IFD were enrolled in this study. **Figure 1** depicts a flow diagram of the study participants based on STARD 2015 (Cohen et al., 2016). All patients received antifungal prophylaxis, according to revised recommendations from a consensus process led by the Gruppo Italiano Trapianto Midollo Osseo (Girmenia et al., 2014). Treatment should be continued for at least 100 days following transplantation, or 180 days in higher-risk patients, or until immunosuppressive therapy is discontinued. Different types of specimens were collected depending on the type of suspected infection. CMT included microscopy, culture, serological tests, RT-PCR, and radiological examinations. NGS tests were performed at Huada Laboratories (Shenzhen, China) or Genskey Laboratories (Tianjin, China). Patients’ antifungal treatment as well as clinical and laboratory data were collected retrospectively at the onset of infection symptoms. The follow-up ended on 31 January 2023.

### 2.2 Diagnosis of IFD

According to the revised definitions of IFD from European Organization for Research on Treatment of Cancer (EORTC) and the Chinese guidelines for the diagnosis and treatment of IFD in patients with hematological disorders and cancers (Chinese Association, H and Chinese Invasive Fungal Infection Working, G, 2020; Donnelly et al., 2020; Alexander et al., 2021), the 46 patients were classified as having proven, probable, possible, or undefined IFD. Patients were defined as having a proven IFD if fungi were discovered in a sterile specimen using cytology, microscopy, or culture. Patients were classified as having probable IFD if a host factor met both clinical criteria (i.e., radiographic findings, bronchoscopy, or sinus analysis) and mycological criteria (i.e., direct detection of fungi in a sterile specimen or detection of specific fungal antigens and cell wall components). Patients were classified as having possible IFD if a host factor met clinical criteria but not mycologic criteria and as having undefined IFD if a host factor did not match both clinical and mycological criteria yet the diagnostic-driven therapy was effective. Positive results for multiple microorganisms in the same specimen were treated as separate events. Overall survival (OS) was measured from the start of HSCT until death or the last follow-up for any reason.

### 2.3 mNGS procedure

#### 2.3.1 Plasma sample processing and DNA extraction

Within 8 h of collection, 3 mL of blood was collected from patients, deposited in a blood collection tube, and held at room temperature for 3 min to 5 min before plasma separation and centrifugation at 4,000 rpm for 10 min at 4°C. Plasma samples were transferred to new sterile tubes. TIANamp Micro DNA Kit (DP316, TIANGEN BIOTECH, Beijing, China) was used to extract DNA from 300 µL of plasma according to the manufacturer’s instructions. The extracted DNA specimens were then utilized to build DNA libraries (Long et al., 2016).

#### 2.3.2 Other samples processing and DNA extraction

BALF/CSF/hydrothorax sample (1.5 mL to 3mL) from patient was collected according to standard procedures. A 1.5-mL microcentrifuge tube containing 0.6 mL of the sample and 250 μL of 0.5-mm enriching beads were attached to a horizontal platform on a vortex mixer and agitated vigorously at 2800–3200 rpm for 30 min. Then, 7.2 μL of lysozyme was added for wall-breaking reaction. The 0.3-mL sample was separated into a new 1.5-mL microcentrifuge tube, and DNA was extracted using the TIANamp Micro DNA Kit (DP316, TIANGEN BIOTECH) according to the manufacturer’s recommendation.

#### 2.3.3 Construction of DNA libraries and sequencing

DNA libraries were constructed through DNA fragmentation, end-repair, adapter-ligation, and PCR amplification. Agilent 2100 (Agilent Technologies, Santa Clara, California) was used for quality control of the DNA libraries. Quality-qualified libraries were pooled, and DNA Nanoball (DNB) was made and sequenced by MGISEQ-200/MGISEQ-2000 platform (Jeon et al., 2014).

#### 2.3.4 Bioinformatic analysis

High-quality sequencing data were generated by removing low-quality reads, followed by computational subtraction of human host sequences mapped to the human reference genome (hg19) using Burrows–Wheeler Alignment (Li and Durbin, 2009). The remaining data by removal of low-complexity reads were classified by simultaneously aligning to Pathogens metagenomics Database, consisting of bacteria, fungi, viruses, and parasites. The classification reference databases were downloaded from National Center for Biotechnology Information (NCBI) (ftp://ftp.ncbi.nlm.nih.gov/genomes/). RefSeq contains 4,945 whole-genome sequence of viral taxa, 6,350 bacterial genomes or scaffolds, 1,064 fungi related to human infection, and 234 parasites associated with human diseases.

#### 2.3.5 Criteria for a positive mNGS result

1. The total number of sample sequences is higher than or equal to 20 million reads.
2. The ratio of the reads per million sample divided by the reads per million of the no-template control from any given taxon (species, genus, or family) ≥10 (Mongkolrattanothai et al., 2017; Simner et al., 2018).
3. Bacteria (mycobacteria excluded), virus, and parasites: mNGS identified a microbe (species level) whose coverage rate scored 10-fold greater than that of any other microbes (Langelier et al., 2018; Miao et al., 2018).
4. Fungi: mNGS identified a microbe (species level) whose coverage rate scored five-fold higher than that of any other fungus because of its low biomass in DNA extraction (Bittinger et al., 2014; Schlaberg et al., 2017; Miao et al., 2018)

### 2.4 Statistical analysis

We used the Kaplan–Meier method for survival analysis, and groups were compared using the log-rank test. The kappa (κ) statistic was used to assess the consistency of different assays. Independent risk factors for IFD were examined using univariate and multivariate logistic regression models. SPSS (version 26.0; SPSS Inc., Chicago, IL, USA), GraphPad Prism (version 8.0; GraphPad Software, La Jolla, CA, USA), and R (version 3.6.3; the R Foundation, Indianapolis, IN, USA) were used to analyze and generate the graphs. *P-*values < 0.05 (two-tailed) were considered statistically significant.

## 3 Results

### 3.1 Clinical characteristics

**Table 1** displays the baseline patient information and baseline characteristics. Forty-six patients with a median age of 43 years were enrolled in this study. The majority of the patients (41.30%) had an underlying diagnosis of AML. Most (89.13%, n = 41) underwent allogeneic HSCT, whereas five (10.87%) underwent autologous HSCT. Eleven (23.91%) had a history of fungal infection. When the symptoms first appeared, 25 (32.2%) of the patients had agranulocytosis, and 36 (78.26%) were given immunosuppressive medication. GVHD occurred in 16 allogeneic HSCT patients, and 35/46 (76.09%) received prophylactic antifungal therapy during IFD. Central venous catheters were placed in 35 patients during the IFD. The median values of LDH, CRP, PCT, and IL-6 in all patients were 339.00 U/L [interquartile range (IQR), 252.50–552.50], 100.20 mg/L (IQR, 42.75–207.88), 0.63 ng/ml (IQR, 0.24–2.37) and 101.20 pg/ml (IQR, 36.53–415.60), respectively.

**Table 1:** Demographics and clinical characteristics of patients.

Characteristic	All patients (n = 46)
Age, years (median, IQR)	43(17.75-52.25)
Male	26(56.50%)
Protopathy	
AA	12(26.09%)
ALL	7(15.22%)
AML	19(41.30%)
MDS	2(4.35%)
MM	3(6.52%)
Lymphoma	3(6.52%)
Transplant-related characteristics	
Donor type	
MRD	30(65.22%)
MSD	9(19.56%)
MUD	2(4.35%)
Auto	3(6.52%)
Auto+CART	2(4.35%)
Absolute counts of CD34+ cells (10^6^/kg)	6.72(4.08-8.00)
N engraftment, days, median (IQR)	14(13-15)
PLT engraftment, days, median (IQR)*	14(13-19.5)
Concomitant conditions^#^	
Central venous line	35(76.08%)
Agranulocytic	25(54.35%)
Immunosuppressive drugs	36(78.26%)
Diabetes	5(10.87%)
Hypoproteinaemia	35(76.09%)
Previous fungal infections	11(23.91%)
Prophylactic antifungal therapy	35(76.09%)
Voriconazole + Sulfamethoxazole	29
Posaconazole + Sulfamethoxazole	6
LDH (U/L), median (IQR)	339.00(252.50-552.50)
CRP (mg/L), median (IQR)	100.20(42.75-207.88)
PCT (ng/ml), median (IQR)	0.63(0.24-2.37)
IL-6 (pg/ml), median (IQR)	101.20(36.53-415.60)
Graft versus host disease	16(34.78%)
Sample collection time	
pre-engraftment period	20(43.48%)
<100D	5(10.87%)
>100D	21(45.65%)
Follow-up time, median (IQR)	16.500 (6.830-32.491)

PLT engraftment, days (median, IQR)*: 1 patient without engraftment was excluded; Concomitant conditions ^#^: Concomitant conditions during infection; AA, Aplastic anemia; AML, Acute myeloid leukemia; ALLL, Acute lymphoblastic leukemia; MDS, Myelodysplastic syndromes; MM, Multiple myeloma; MRD, Mismatched related donor; MSD, Matched sibling donor; MUD, Matched unrelated donor; Auto, Autologous; CART, Chimeric Antigen Receptor T-Cell Immunotherapy; IQR, Interquartile range.

### 3.2 IFD diagnoses

**Figure 2:**
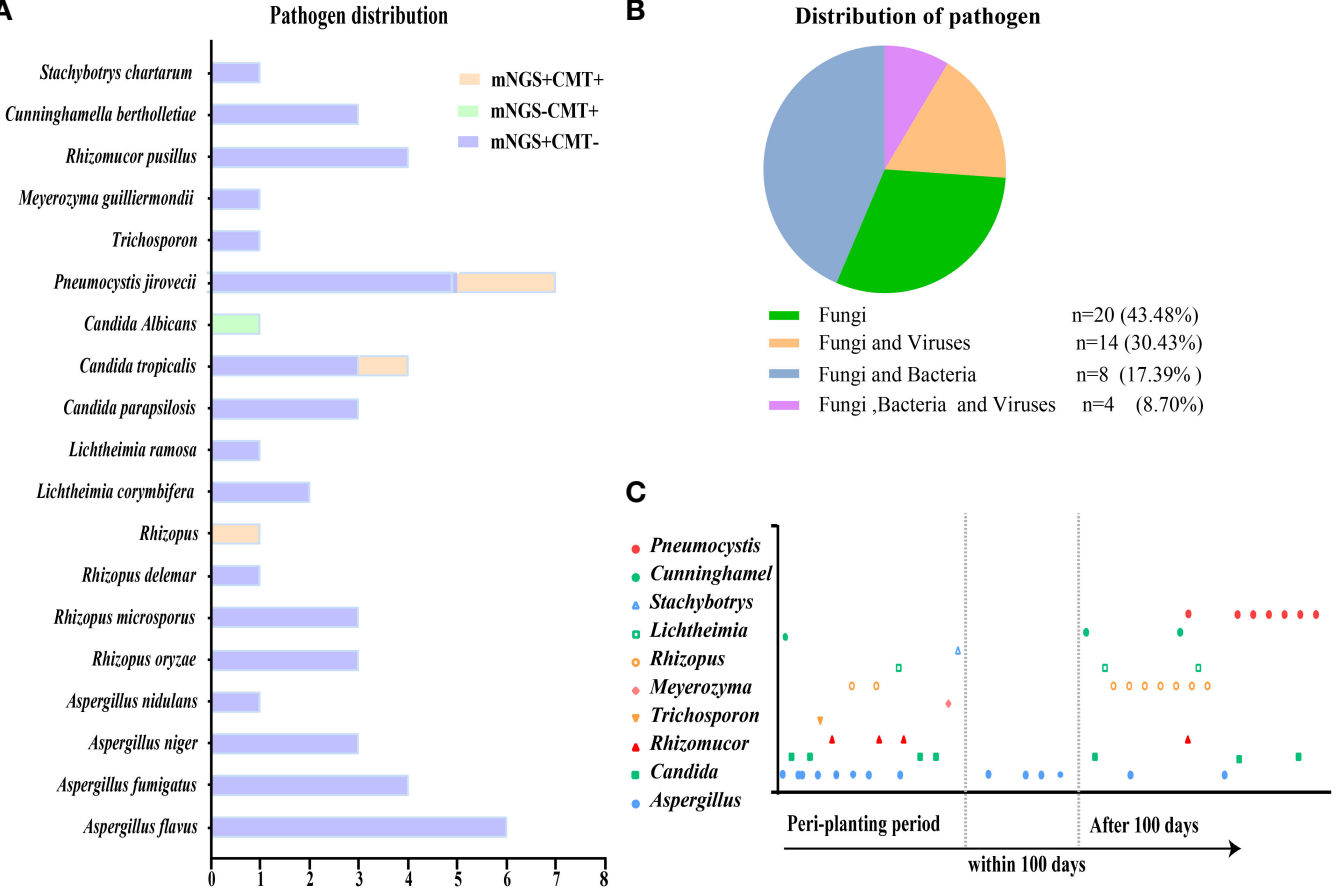
Distribution of pathogens identified in patients with IFD after HSCT. **(A)** The figure showed the number of subjects in whom each causative fungus was detected. Orange bars indicate fungi detected by both CMT and mNGS (mNGS+CMT+). Purple bars indicate fungi detected by mNGS only (mNGS+CMT−). Green bars indicate fungi detected only by CMT (mNGS−CMT+). **(B)** Distribution of pathogens was shown in patients. Fungal infections accounted for 43.48% among all the subjects. **(C)** Different fungal infections occurred at different periods after transplantation.

In total, 46 patients were diagnosed with IFD, and the prevalence of proven, probable, possible, and undefined IFD was 2.18% (1), 23.91% (11), 58.69% (27), and 15.22% (7), respectively. Candida tropicalis was cultured from the peripheral blood of a patient with proven IFD. **Figure 2A** depicts the distribution of pathogens detected using mNGS and CMT. The most common IFD was *Aspergillosis*, which accounted for 30.43% of the episodes (14); *Candidiasis* and *Pneumocystis* each accounted for 15.22% of the episodes (7). One of whom had *Candidiasis* and *Aspergillosis*, and another had *Candidiasis* and *Pneumocystis*. Fifteen patients were diagnosed with *mucormycosis*, three of whom had *Aspergillus* infection and one had *Candida* infection. *Trichosporon*, *Meyerozyma*, and *Stachybotrys* were detected in one case each. The remaining six cases of IFD were diagnosed without formal identification of fungal species, for example, by BDG. mNGS outperformed other methods in detecting fungi and accurately detected specific fungal pathogens. The majority (56.52%) of patients with IFD were co-infected with bacteria and/or viruses (**Figure 2B**). **Figure 2C** displays the pathogen distribution in 46 patients with IFD at different times following transplantation. A total of 57.14% of the *Aspergillus* infections in our study occurred during the peri-planting period. All *Pneumocystis* infections occurred later than 100 days after transplantation.

### 3.3 Diagnostic performance of mNGS compared to CMT

**Figure 3:**
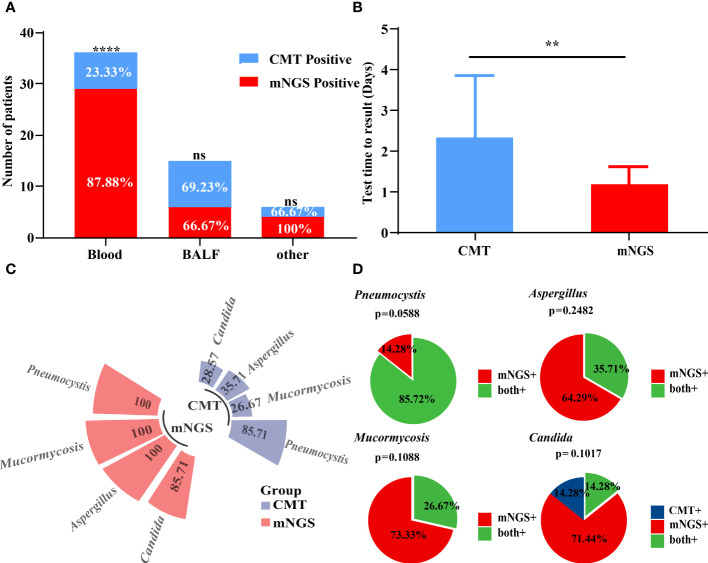
Performance of mNGS and CMT in pathogens identified. **(A)** The positive rates of mNGS and CMT in different samples were compared. The positive rate of mNGS in peripheral blood samples was significantly higher than that of CMT. **(B)** The diagnostic time required for mNGS and CMT were compared in patients with IFD. **(C, D)** The positive detection rate of mNGS and CMT and their consistency in different pathogens. **P < 0.01 and ****P < 0.0001 by Wilcoxon rank sum test.

The sensitivity of both mNGS and CMT in all patients is shown in **Table 2**. Our results showed that mNGS and CMT had a sensitivity of 84.78% and 36.96%, respectively. mNGS significantly outperformed CMT (*P* < 0.0001). Fifteen (32.61%) of the 46 patients had congruent mNGS and CMT results. Additionally, sample selection is essential for testing performance. The sensitivity rates of mNGS and CMT for different specimens are shown in **Figure 3A**. The sensitivity of peripheral blood samples to mNGS was significantly higher (*P* < 0.0001), but, for specimens from potentially pathological tissues, such as sputum and bronchoalveolar lavage fluid (BALF) from patients with cough, the sensitivity rates of mNGS and CMT were comparable. When compared to CMT, mNGS significantly reduced the time needed to identify pathogens (*P* = 0.0016) (**Figure 3B**). mNGS had a higher positive testing % for the detection of various pathogenic fungi than CMT. The consistency for *Pneumocystis* was 85.72% (**Figures 3C, D**).

**Table 2:** Performance of mNGS and CMT in all patients with IFD.

	mNGS		
CMT	Positive	Negative	Total
Positive	15	2	17
Negative	24	5	28
Total	9	7	46

### 3.4 Antifungal therapy

All patients received prophylactic antifungal therapy. When symptoms appeared, 35/46 (76.09%) patients received prophylactic antifungal therapy, including 29 patients who received voriconazole plus sulfamethoxazole and six patients who received posaconazole plus sulfamethoxazole. However, 71.43% of the patients with *Aspergillus* infection had breakthrough infection after starting voriconazole, and intravenous voriconazole was substituted in the context of routine drug monitoring. Of these, 78.75% received two or more antifungal drugs in subsequent treatment. In addition, 71.43% of *pneumocystis* infections occurred withrow the sulfamethoxazole, and all patients were still effectively treated in the subsequent therapy using sulfamethoxazole in combination with or without other antifungal drugs. At the time of infection, 12 of the 15 (80%) patients with *mucormycosis* received prophylactic antifungal therapy, with nine patients receiving voriconazole + sulfamethoxazole and the remaining three receiving posaconazole + sulfamethoxazole. Fourteen of the 15 patients (93.3%) with *mucormycosis* subsequently received posaconazole or amphotericin B in combination with other antifungal drugs. Two of them had undergone surgery, and one patient, according to mNGS results, was not treated with appropriate antifungal drugs in time, resulting in disease progression and death. All patients with *Candida* infection had the central venous line removed and received antifungal therapy. *Posaconazole* in combination with amphotericin B was administered to one patient with *Trichosporon* infection and another with *Meyerozyma* infection. The patient with *Stachybotrys* infection was treated with amphotericin B and *pneumocystis*. Based on the NGS results, dose adjustments or medication changes using empiric antifungal treatment occurred in 56.52% of patients with IFD. Details of the antifungal treatments are described in **Table 3**. Such high values may be attributed to the combination of antifungal therapies.

**Table 3:** Antifungal therapy and mortality in IFD patients after HSCT.

	N	The period of symptoms occurs (N)		Prophylactic antifungal therapy		Antifungal therapy				Outcome (N)	
		≤100 d	>100 d	Regimen 1 (N)	Regimen 2 (N)	Regimen 1	Regimen 2	Regimen 3	Regimen 4	Improved	Death
*Aspergillus*											
Probable	1	0	1	POS+SOX (1)		VOR				1	0
Possible	5	5	0	VOR+SOX (5)		VOR	ABCD+VOR	VOR+AMB		4	1
Undefined	4	4	0	VOR+SOX (4)		VOR	AmBisome + VOR			4	0
*Mucor*^1^											
Probable	2	1	1	VOR+SOX (2)		POS+AMB	ABCD			1	1
Possible	9	4	5	VOR+SOX (5)	POS+SOX (2)	POS	ABCD	AmBisome+POS	MIF+VOR	6	3
*Candida*^2^											
Proven	1	1	0	VOR+SOX (1)		CAS				1	0
Probable	1	0	1	VOR+SOX (1)		ABCD+VOR				1	0
Possible	1	1	0	VOR+SOX (1)		CAS				1	0
Undefined	1	1	0	POS+SOX (1)		CAS+ ABCD				1	0
*Pneumocystis*											
Probable	4	0	4	VOR+SOX (2)		SOX				4	0
Possible	1	0	1			SOX				1	0
Undefined	1	0	1			SOX				1	0
Mixed pathogenic fungi											
Probable	1	0	1	VOR+SOX (1)		ABCD+VOR				1	0
Possible	4	2	2	VOR+SOX (1)		AmBisome+ VOR+CAS	VOR+CAS	AMB+POS	SOX+VOR+CAS	2	2
Undefined	1	1	0	POS+SOX (1)		ABCD+MIF				1	0
Others											
Probable	2	2	0	VOR+SOX (1)		ABCD, AMB				2	0
Possible	7	4	3	VOR+SOX (5)	POS+SOX (1)	AMB+POS	VOR	MIF+POS	AMB+VOR	6	1

1. Two patients had been treated with surgical procedure; 2. All patients had removed central venous line.

POS, posaconazole; VOR, voriconazole; SOX, sulfamethoxazole; AMB, amphotericin B; CAS, caspofungin; MIF, micafungin; AmBisome, liposomal amphotericin B; ABCD, amphotericin B cholesteryl sulfate.

### 3.5 Outcomes and survival

**Figure 4:**
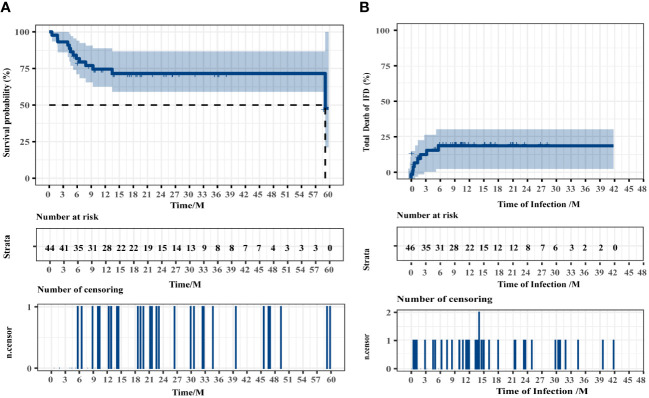
Overall survival of all patients **(A)** and the patients who died of IFD **(B)**. Two patients were excluded from the overall survival analysis because of intracranial hemorrhage and disease recurrence during conditioning.

The median follow-up time was 16.50 months (range, 0.57–59.79 months). The estimated 4-year OS of patients with IFD was 71.55% (95% CI, 55.18%–85.82%), and the median survival time (mOS) was 59.07 months (95% CI, 55.30–62.84 months). The median time from fungal infection to death or the end of follow-up was 11.79 months. The fungi-related mortality rate was 18.82% (95% CI, 9.08%–30.72%). The survival data of patients with IFD are presented in **Figure 4**. Multivariate logistic regression analyses were performed for all 189 patients to identify the independent risk factors for IFD following HSCT and to correct for potential confounding factors. Univariate analysis was used to select variables for the multivariate logistic regression studies. Independent risk factors for IFD (**Table 4**) included corticosteroid use (Hazard ratio (HR), 8.008; 95% CI, 3.550–18.063; *P* < 0.001) and hypoproteinemia (HR, 5.121; 95% CI, 2.245–11.681; *P* < 0.001).

**Table 4:** Univariate and multivariate analyses for IFD.

	Univariate analysis		
	*P-*value	HR	95% CI
Age (years)	0.015	1.025	1.005–1.046
Male gender	0.818	1.082	0.553–2.118
Diabetes	0.232	0.490	0.152–1.579
Prior fungal infection	0.026	2.661	1.122–6.309
Agranulocytic	0.498	1.259	0.646–2.454
Corticosteroid	<0.001	6.231	2.910–13.344
Immunosuppressive drugs	0.833	1.090	0.489–2.429
Monoclonal antibodies	0.373	1.484	0.623–3.536
Central venous catheterization	0.459	1.336	0.620–2.879
Unrelated donor	0.359	1.820	0.506–6.551
Haplo	0.573	0.819	0.409–1.630
GVHD	0.255	1.514	0.742–3.088
CMV	0.503	1.278	0.623–2.621
Hypoproteinemia	<0.001	9.369	4.313–20.353
	Multivariate analysis		
	*P-*value	HR	95% CI
Corticosteroid	<0.001	8.008	3.550–18.063
Hypoproteinemia	<0.001	5.121	2.245–11.681

HR, hazard ratio; CI, confidence interval.

## 4 Discussion

IFD is a common complication of HSCT that is associated with high morbidity and mortality rate (Neofytos et al., 2009; Sun et al., 2015). Although IFD treatment has advanced significantly, delayed diagnosis causes significant morbidity and mortality (Ostrosky-Zeichner, 2012). Substantial advances in the diagnostic approaches for IFD include radiographic findings, biomarkers (e.g., GM and BDG), and PCR assays (Greene et al., 2007; Arvanitis et al., 2015). However, CMT may not be appropriate for rare fungi, particularly those that are difficult to culture, resulting in delayed detection, whereas mNGS can directly execute DNA or RNA sequencing (Tsang et al., 2021). Jiang et al. (2022) report that mNGS is increasingly being used to treat fungal infections.

The predominant fungal species in our study were *Aspergillus* (14 of 46, 30.43%) and *Rhizopus* (8 of 46, 17.39%). *Candida* and *Pneumocystis* accounted for 15.22% (7 of 46) of the infections. Owing to the different characteristics of immune function damage, the pathogens of IFD also differ at different stages after transplantation. Twenty (43.48%) of the 46 patients developed IFD during the conditioning period to the engraftment period, five (10.87%) within 100 days after transplantation, and 21 (45.65%) cases occurred more than 100 days later.

In our study, mNGS technology had distinct advantages over CMT in diagnosing different types of fungal species. The detectability increased significantly from 36.96% to 84.78%. The detection time was significantly reduced using mNGS. mNGS also provides accurate identification of specific fungal pathogens and is more specific than CMT. The mNGS results for 15 patients were consistent with those of CMT. The quick and accurate diagnosis of fungal species is critical for selecting appropriate antifungal agents, avoiding resistance, and managing patients (Omrani and Almaghrabi, 2017; Wang et al., 2020; Stemler et al., 2022). Pathogens are distributed in a variety of tissues and organs and have the ability to detect variations in different types of samples. The sensitivity of peripheral blood samples to CMT, particularly blood cultures, was significantly lower than that of mNGS; however, for specimens from possible pathological tissues, the sensitivity of mNGS and CMT was comparable.

In two incidents the CMT results in this study were positive, but the NGS produced a false negative in two patients. The specific analysis was as follows: mNGS detected human beta-herpesvirus 5 and torque teno virus in P1, whereas blood culture detected *Candida albicans*. Further analysis of the original mNGS data revealed that mNGS detected *Candida albicans* using an updated database. The second incident involved P6, where a positive GM test in the BALF was accompanied by an abnormal chest CT scan, which improved after receiving antifungal therapy.

Sometimes, the CMT results can indicate that the patient experienced suspected IFD following HSCT; however, the identification of fungal species is nonspecific. mNGS has low sensitivity when: 1) the pathogen load is below the detection limit or is filtered owing to low-ranking readings; 2) the pathogen’s nucleic acid is easily degraded; 3) the pathogen is suspected to be a background microorganism; and 4) the dataset or database has limitations. Further reasonable interpretations of the results are then required. The mcfDNA-Seq assay and serum GM can generally provide complementary results for the diagnosis of invasive pulmonary mold infections following HSCT (Hill et al., 2021). As proposed for *P. jirovecii* infection following HSCT (Liu et al., 2021), mNGS and BDG may aid in distinguishing colonization from infection. By using CMT in conjunction with mNGS technologies, the vision of a “one-stop” for IFD diagnosis seems promising in the foreseeable future.

Despite a decline in IFD-related deaths over the past decade, IFD remains a significant limiting factor for effective HSCT (Neofytos et al., 2009; Kontoyiannis et al., 2010). As a result, patients with suspected IFD after HSCT are frequently given prophylactic or empirical antifungal treatments. Most of the patients (76.09%) in our study received prophylactic antifungal therapy, with voriconazole being the most widely used drug. Although most patients received antifungal prophylaxis, many patients developed GVHD or required corticosteroids and immunosuppressants following HSCT. Among the patients with *Pneumocystis* infection, 71.43% occurred withrow the sulfamethoxazole, and all patients were still successfully treated in the subsequent therapy with sulfamethoxazole in combination with or without other antifungal drugs. This suggests that prophylactic antifungal therapy lowers the risk of IFD. According to the NGS results, dose adjustments or medication changes on the basis on empiric antifungal treatment, occurred in 56.52% of patients with IFD. The estimated 4-year OS was 71.55%, whereas the mOS of patients was 59.07 months. Eight patients died because of IFD. In this investigation, corticosteroid use (HR, 8.008; 95% CI, 3.550–18.063) and hypoproteinemia (HR, 5.121; 95% CI, 2.245–11.681) were identified as independent risk factors for IFD using multivariate logistic regression.

This study has several limitations. First, being a retrospective study, there were some inherent limitations, such as information bias and selection bias. The sample size of our study was relatively small. In addition, mNGS was usually performed only once owing to its high cost. Finally, there was no information on antifungal susceptibility or resistance. Further prospective randomized controlled studies with larger sample sizes are needed to be designed to elucidate the diagnostic value of mNGS for IFD following HSCT according to STARD 2015.

In conclusion, our study demonstrated the feasibility of mNGS in patients with IFD following HSCT. We recommend that clinicians use mNGS technology more actively when there is a clinical suspicion of IFD after HSCT. For clinical infection related samples, the traditional microbiological detection should be improved first. Pathology and sterile specimen culture are still the gold standard for infection diagnosis. Pathogenic mNGS is a powerful supplement and extension, which may be a helpful adjunct to the clinical diagnosis of IFD, allowing for more rapid and focused antifungal treatment.

## Data availability statement

The datasets presented in this study can be found in online repositories. The name of the repository and accession number can be found below: NCBI: PRJNA1085906.

## Author contributions

YZ and NW designed, supervised the clinical study, and revised the manuscript, who contributed equally to this work and should be considered corresponding authorship. XZ analyzed data and drafted the manuscript. LZ collected clinical data. YL was responsible for patient management. All authors contributed to the article and approved the submitted version.
