# Supplementary material for: Clinical performance of metagenomic next-generation sequencing for diagnosis of invasive fungal disease after hematopoietic cell transplant
Source: Front Cell Infect Microbiol. 2024 Mar 25;14:1210857. doi: 10.3389/fcimb.2024.1210857 (PMC11000502; doi:10.3389/fcimb.2024.1210857)
Supplement: Supplementary file 1 [file DataSheet_1.pdf]

Supplementary Material

Clinical Performance of Metagenomic Next-Generation Sequencing for Diagnosis of Invasive fungal disease after Hematopoietic Cell Transplant

Xiaoying Zhang[1], Lifeng Zhang[1], Yun Li[1], Na Wang[1*] and Yicheng Zhang[1,2, 3*]
Correspondence:

Corresponding Author:

Yicheng Zhang,
yczhang@tjh.tjmu.edu.cn;

Na Wang,

wangna.2001@163.com

Supplementary method

Protocol of conventional microbiological test

1) Fungal smear and culture:

Gram stain, KOH test, and Ziehl-Neelsen stain were used to identify bacteria, fungi and Mycobacterium tuberculosis complex (MTBC) by smear microscopy. Samples were inoculated onto blood culture bottles. Simultaneously, the blood culture bottles were incubated in the BACTEC FX400 automated blood culture system until positivity or for a maximum of 5 days. The susceptibility testing was performed by disc diffusion according to the EUCAST method. Sabouraud dextrose agar supplemented with and without chloramphenicol were used to isolate fungi at 28 and 35◦C for up to 5 days, respectively. All cultured microorganisms were identified using the Bruker MALDI Biotyper (Bruker Corporation, Germany). Filamentous fungi were identified according to colony morphology and smear results;

2) 1,3-β-D glucan test (G test) and galactomannan antigen detection (GM test)

Microplate double antibody sandwich method was used for detection. GM test detects glactomannan (GM), which is mainly suitable for the early diagnosis of invasive aspergillosis. GM is a kind of polysaccharide widely present in the cell wall of Aspergillus and Penicillium. 1, 3-B-D glucan is a fungal cell wall component, which

is suitable for the early diagnosis of all deep fungal infections except cryptococcus and zygomycetes (mucor), especially Candida and Aspergillus, but it can not determine the species.

3)Real-time PCR:

The RT-PCR were performed by available commercial kits. Total cell RNA was isolated, and 100 ng of each RNA sample was reverse transcribed and assayed by RT-PCR. Transcript quantification was performed with NMPA approved commercial kits. Duplicates were run for each sample in a 96-well plate. β-Actin was used as the endogenous reference gene. All quantitative RT-PCR reactions were run in three independent experiments. The relative quantification method was used, with the ratio of the mRNA level for the gene of interest normalized to the level of β-actin and the mean of control samples as the calibrator value. The specificity of the products was confirmed based on melting.

Supplementary Tables

Supplementary Table 1. Criteria for the diagnostic class of IFD and corresponding antifungal therapeutic strategy[1, 2].

Elements	FN	Undefined IFD	Undefined IFD	Undefined IFD	Possible IFD	Probable IFD	Proven IFD[a]
Host factors	+	+	+	+	+	+	/
Clinical and radiological manifestations	No	No	Uncharacteristic	Uncharacteristic	Characteristic	Characteristic	/
Microbiology laboratory examinations（G/GM）	Negative	Negative	Negative	Positive	Negative	Positive	/
Criteria for proven invasive fungal disease	Nonconforming	Nonconforming	Nonconforming	Nonconforming	Nonconforming	Nonconforming	Conforming
Clinical criteria of IFD	Nonconforming	Nonconforming	Nonconforming	Nonconforming	Conforming	Conforming	/
Antifungal therapy	empiric therapy	Diagnostic-driven therapy	Diagnostic-driven therapy	Diagnostic-driven therapy	Diagnostic-driven therapy	Targeted Therapy	Targeted Therapy

Note: IFD, Invasive Fungal Disease; G, (1,3)-β-D-glucan testing; GM, galactomannan antigen testing； “/”, Not required；

[a] Proven IFD is independent of host factor, Clinical and radiological manifestations

Supplementary Table 2. Criteria for proven invasive fungal disease except for endemic mycoses[1, 2]

Analysis and specimen	Molds[a]	Yeasts[a]
Microscopic analysis: sterile material	Histopathologic, cytopathologic, or direct microscopic examination[b] of a specimen obtained by needle aspiration or biopsy in which hyphae or melanized yeast-like forms are seen accompanied by evidence of associated tissue damage	Histopathologic, cytopathologic, or direct microscopic examination[b] of a specimen obtained by needle aspiration or biopsy from a normally sterile site (other than mucous membranes) showing yeast cells—for example, Cryptococcus species indicated by encapsulated budding yeasts or Candida species showing pseudohyphae or true hyphae[c]
**Culture**		
Sterile material	Recovery of a mold or “black yeast” by culture of a specimen obtained by a sterile procedure from a normally sterile and clinically or radiologically abnormal site consistent with an infectious disease process, excluding bronchoalveolar lavage fluid, a cranial sinus cavity specimen, and urine	Recovery of a yeast by culture of a sample obtained by a sterile procedure (including a freshly placed [<24 h ago] drain) from a normally sterile site showing a clinical or radiological abnormality consistent with an infectious disease process
Blood	Blood culture that yields a mold[d] (e.g., Fusarium species) in the context of a compatible infectious disease process	Blood culture that yields yeast (e.g., Cryptococcus or Candida species) or yeast-like fungi (e.g., Trichosporon species)
Serological analysis: CSF	Not applicable	Cryptococcal antigen in CSF indicates disseminated cryptococcosis

[a] If culture is available, append the identification at the genus or species level from the culture results;

[b] Tissue and cells submitted for histopathologic or cytopathologic studies should be stained by Grocott-Gomorri methenamine silver stain or by periodic acid Schiff stain, to facilitate inspection of fungal structures. Whenever possible, wet mounts of specimens from foci related to invasive fungal disease should be stained with a fluorescent dye (e.g., calcofluor or blankophor);

[c] Candida, Trichosporon, and yeast-like Geotrichum species and Blastoschizomyces capitatus may also form pseudohyphae or true hyphae.

Supplementary Table 3. Criteria for probable invasive fungal disease except for endemic mycoses[1, 2]

Host factors [a]

Recent history of neutropenia ($<0.5 \times 10^9$ neutrophils/L [<500 neutrophils/mm^3] for >10 days) temporally related to the onset of fungal disease

Hematologic malignancy

Receipt of an allogeneic stem cell transplant

Solid organ transplant recipient

Prolonged use of corticosteroids (excluding among patients with allergic bronchopulmonary aspergillosis) at a therapeutic dose of ≥0.3 mg/kg corticosteroids for ≥3 weeks in the past 60 days

Treatment with other recognized T-cell immunosuppressants, such as calcineurin inhibitors, tumor necrosis factor-a blockers, lymphocyte-specific monoclonal antibodies, immunosuppressive nucleoside analogues during the past 90 days

Inherited severe immunodeficiency (such as chronic granulomatous disease, STAT 3 deficiency, CARD9 deficiency, STAT-1 gain of function, or severe combined immunodeficiency)

Acute graft-versus-host disease grade III or IV involving the gut, lungs, or liver that is refractory to first-line treatment with steroids

Clinical criteria [b]

Lower respiratory tract fungal disease[c]

The presence of 1 of the following 3 signs on CT:

Dense, well-circumscribed lesions(s) with or without a halo sign

Air-crescent sign

Cavity

Tracheobronchitis

Tracheobronchial ulceration, nodule, pseudomembrane, plaque, or eschar seen on bronchoscopic analysis

Sinonasal infection

Imaging showing sinusitis plus at least 1 of the following 3 signs:

Acute localized pain (including pain radiating to the eye)

Nasal ulcer with black eschar

Extension from the paranasal sinus across bony barriers, including into the orbit

Extension from the paranasal sinus across bony barriers, including into the orbit

1 of the following 2 signs:

Focal lesions on imaging

Meningeal enhancement on MRI or CT

Disseminated candidiasis [d]

At least 1 of the following 2 entities after an episode of candidemia within the previous 2 weeks:

Small, target-like abscesses (bull's-eye lesions) in liver or spleen

Progressive retinal exudates on ophthalmologic examination

Mycological criteria

Direct test (cytology, direct microscopy, or culture)

Mold in sputum, bronchoalveolar lavage fluid, bronchial brush, or sinus aspirate samples, indicated by 1 of the following:

Presence of fungal elements indicating a mold

Recovery by culture of a mold (e.g., Aspergillus, Fusarium, Zygomycetes, or Scedosporium species)

Indirect tests (detection of antigen or cell-wall constituents) [e]

Aspergillosis: Galactomannan antigen detected in plasma, serum, bronchoalveolar lavage fluid, or CSF

Invasive fungal disease other than cryptococcosis and zygomycoses: β-d-glucan detected in serum

NOTE. Probable IFD requires the presence of a host factor, a clinical criterion, and a mycological criterion. Cases that meet the criteria for a host factor and a clinical criterion but for which mycological criteria are absent are considered possible IFD.

[a] Host factors are not synonymous with risk factors and are characteristics by which individuals predisposed to invasive fungal diseases can be recognized. They are intended primarily to apply to patients given treatment for malignant disease and to recipients of allogeneic hematopoietic stem cell and solid-organ transplants. These host factors are also applicable to patients who receive corticosteroids and other T cell suppressants as well as to patients with primary immunodeficiencies；

[b] Must be consistent with the mycological findings, if any, and must be temporally related to current episode；

[c] Every reasonable attempt should be made to exclude an alternative etiology；

[d] The presence of signs and symptoms consistent with sepsis syndrome indicates acute disseminated disease, whereas their absence denotes chronic disseminated disease；

[e] These tests are primarily applicable to aspergillosis and candidiasis and are not useful in diagnosing infections due to Cryptococcus species or Zygomycetes (e.g., Rhizopus, Mucor, or Absidia species). Detection of nucleic acid is not included, because there are as yet no validated or standardized methods

References

[1] DONNELLY J P, CHEN S C, KAUFFMAN C A, et al. Revision and Update of the Consensus Definitions of Invasive Fungal Disease From the European Organization for Research and Treatment of Cancer and the Mycoses Study Group Education and Research Consortium [J]. Clinical Infectious Diseases, 2020, 71(6): 1367-76.

[2] CHINESE ASSOCIATION H, CHINESE INVASIVE FUNGAL INFECTION WORKING G. [The Chinese guidelines for the diagnosis and treatment of invasive fungal disease in patients with hematological disorders and cancers (the 6th revision)] [J]. Zhonghua Nei Ke Za Zhi Chinese Journal of Internal Medicine, 2020, 59(10): 754-63.
